# Self-Assembly of Molecular Brushes with Polyimide Backbone and Amphiphilic Block Copolymer Side Chains in Selective Solvents

**DOI:** 10.3390/polym12122922

**Published:** 2020-12-05

**Authors:** Maria Simonova, Ivan Ivanov, Tamara Meleshko, Alexey Kopyshev, Svetlana Santer, Alexander Yakimansky, Alexander Filippov

**Affiliations:** 1 Institute of Macromolecular Compounds of the Russian Academy of Sciences, Bolshoy Pr. 31, 199004 Saint Petersburg, Russia; gangspil@gmail.com (I.I.); meleshko@hq.macro.ru (T.M.); yakimansky@yahoo.com (A.Y.); afil@imc.macro.ru (A.F.); 2 Institute of Physics and Astronomy, University of Potsdam, 14476 Potsdam, Germany; kopyshev@uni-potsdam.de (A.K.); santer@uni-potsdam.de (S.S.)

**Keywords:** molecular brushes, amphiphilic side chains, molecular hydrodynamics and optics, conformational and hydrodynamic characteristics, aggregation

## Abstract

Three-component molecular brushes with a polyimide backbone and amphiphilic block copolymer side chains with different contents of the “inner” hydrophilic (poly(methacrylic acid)) and “outer” hydrophobic (poly(methyl methacrylate)) blocks were synthesized and characterized by molecular hydrodynamics and optics methods in solutions of chloroform, dimethylformamide, tetrahydrofuran and ethanol. The peculiarity of the studied polymers is the amphiphilic structure of the grafted chains. The molar masses of the molecular brushes were determined by static and dynamic light scattering in chloroform in which polymers form molecularly disperse solutions. Spontaneous self-assembly of macromolecules was detected in dimethylformamide, tetrahydrofuran and ethanol. The aggregates size depended on the thermodynamic quality of the solvent as well as on the macromolecular architectural parameters. In dimethylformamide and tetrahydrofuran, the distribution of hydrodynamic radii of aggregates was bimodal, while in ethanol, it was unimodal. Moreover, in ethanol, an increase in the poly(methyl methacrylate) content caused a decrease in the hydrodynamic radius of aggregates. A significant difference in the nature of the blocks included in the brushes determines the selectivity of the used solvents, since their thermodynamic quality with respect to the blocks is different. The macromolecules of the studied graft copolymers tend to self-organization in selective solvents with formation of a core–shell structure with an insoluble solvophobic core surrounded by the solvophilic shell of side chains.

## 1. Introduction

Modern methods of polymer synthesis provide great opportunities to obtain macromolecules with a complex architecture such as molecular brushes, star-shaped polymers, hyperbranched polymers or dendrimers, that is, systems containing blocks or components of different chemical nature [1,2,3,4,5]. These systems differ from linear polymers in a number of important physicochemical properties. In particular, the processes of self-organization in their solutions are the subject of intensive research [6,7,8,9,10,11,12].

Among the polymers with a complex architecture, molecular brushes received a significant amount of attention due to their shape and unique properties [13,14,15,16]. The developed synthetic approaches based on “living”/controlled radical polymerization make it possible to obtain graft copolymers with a predetermined molecular architecture, chemical nature of components and controlled molar mass, composition and functionality [15].

Macromolecular brushes with different nature of the backbone and side chains are of special interest because they may adopt a wide variety of conformations in selective solvents [17,18,19]. Their solution behavior depends not only on the parameters of the architecture, primarily on the grafting density and the length of the side chains [6,12], but is influenced significantly by the different thermodynamic quality of the solvent with respect to the backbone and side chains. The ability of brushes to self-organize macromolecules in a selective solvent and the sensitivity of self-assembled structures to external factors provide wide possibilities for their use in various fields, such as catalysis and biotechnology. It is known that micellization of diphilic copolymers in selective solvents is influenced by the thermodynamic quality of the solvent relative to the individual blocks of the copolymer, the molar mass characteristics of the copolymer and its components. The molecular architecture of brushes strongly affects the assembly behavior of macromolecules compared to linear diphilic block copolymers [20,21,22,23,24,25,26,27], which is also largely determined by the spatial arrangement of side chains and lengths of their blocks.

Multicomponent molecular brushes with amphiphilic block copolymer side chains are promising objects. In fact, such systems are ternary block copolymers of complex architecture, which combine three blocks differing in their chemical structure. Such diphilic graft copolymers possess a unique complex of properties due to the ability to form ordered micellar structures by the self-organization of macromolecules in selective solvents [19]. The self-assembly behavior of molecular brushes and the sensitivity of the resulting structures to external factors have gained considerable interest because of the wide possibility of their use as systems for targeted drug delivery [28,29,30] and molecular templates or nanoreactors for the synthesis of organic [31,32,33] and organo-silica hybrid nanotubes and nanowires [34,35]. Molecular brushes with polyelectrolyte diblock copolymer side chains are promising for the synthesis of noble metal nanoparticles (Au and Pt) [36,37,38,39] and materials with magnetic properties [40,41,42].

Previously, molecular brushes with a hydrophobic polyimide (PI) backbone and amphiphilic diblock copolymer side chains of poly(methacrylic acid)-*b*-poly(methyl methacrylate) (PMAA-*b*-PMMA) were synthesized by the ATRP method [43]. The pathways for the controlled regulation of the length and grafting density of hydrophilic and hydrophobic blocks of PMAA and PMMA in the side chains have been established. These copolymers have attracted considerable interest since they provide greater control over the conformation of macromolecules in selective solvents, which is caused by the combination of polyheteroarylene polyimide blocks and amphiphilic polymethacrylate chains in the same macromolecule. In particular, it was shown that they form almost monodisperse micelle-like nanostructures in ethanol [43].

This work is a continuation of previous research and aims to systematically study the behavior of molecular brushes with a polyimide backbone and amphiphilic block copolymer side chains in selective solvents and to establish the effect of the architecture and structural parameters of macromolecules on their conformational characteristics and self-assembly behavior. One of the important tasks is to analyze the influence of the ratio of hydrophilic and hydrophobic blocks in the side chains on the self-organization of three-component brushes in solutions.

## 2. Materials and Methods

### 2.1. Synthesis and Structure Characterization of Molecular Brushes

The synthesis of the investigated copolymers and their characterization were described in detail previously [43]. The target molecular brushes with a polyimide (PI) backbone and block copolymer side chains were synthesized by the “grafting from” approach in conjunction with the ATRP method in several stages through the intermediate formation of molecular brushes with regularly grafted side chains of poly(*tert*-butyl methacrylate) (PtBMA) followed by chain extension of methyl methacrylate (MMA) from living chain ends of PI-*g*-PtBMA. Amphiphilic molecular brushes with hydrophilic blocks of poly(methacrylic acid) (PMAA) in side chains were obtained by selective acidic hydrolysis of PtBMA blocks in side chains of the prepolymer. A description of the materials used and their purity are described in Supplementary Materials.

In order to obtain molecular brushes, samples of the multicenter polyimide macroinitiator with 2-bromoisobutyrate initiating groups in each repeat unit were used. The conversion of monomers (*tert*-butyl methacrylate and methyl methacrylate) was characterized by gas chromatography using a Shimadzu GC-2010 Plus gas chromatograph equipped with a flame ionization detector and an Agilent J&W DB-WAX GC Capillary Column (30 m × 0.53 mm, 0.50 μm film thickness). ^1^H NMR spectra of polymerization products were recorded using a Bruker AC200 (200.1 MHz) spectrometer with DMSO-d_6_ or CDCl_3_ as solvents.

**Figure 1:**
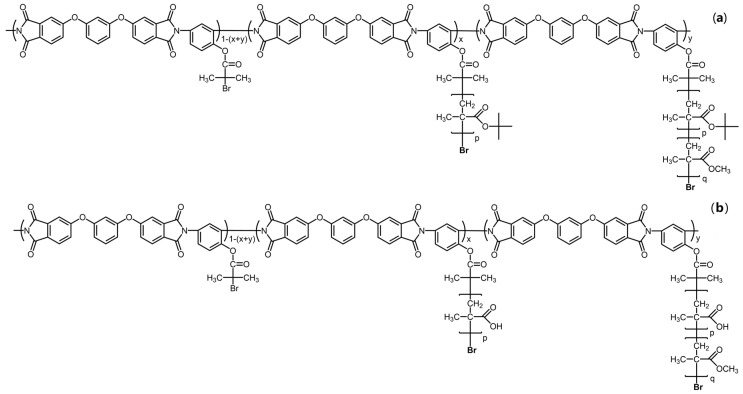
Structure of molecular brushes PI-*g*-(PtBMA-*b*-PMMA) (**a**) and PI-*g*-(PMAA-*b*-PMMA) (**b**) with block copolymer side chains. PI: polymide; PtBMA: poly(*tert*-butyl methacrylate); PMMA: poly(methyl methacrylate); PMAA: poly(methacrylic acid).

The structures of molecular brushes with a hydrophobic polyimide backbone and block copolymer side chains consisting of inner poly(*tert*-butyl methacrylate) or poly(methacrylic acid) and outer poly(methyl methacrylate) blocks are given in Figure 1.

In order to determine the reliable structural parameters of the studied copolymers, the homo- (PtBMA) and block copolymer (PtBMA-*b*-PMMA) side chains were cleaved from the backbone using the selective alkaline hydrolysis described earlier in [44]. Molar mass characteristics of the polyimide macroinitiator and cleaved side chains were analyzed by SEC using an Agilent-1260 Infinity complex equipped with 2 × PLgel MIXED-C columns (7.5 × 300 mm; a particle size of 5 μm) under an isocratic elution regime. DMF containing 0.1 mol/L LiBr was used as an eluent at a flow rate of 1 mL/min and a temperature of 50 °C. Molar mass characteristics of samples were determined from the combined data of the refractometric, viscosimetric and light-scattering detectors. This combination made it possible to avoid application of calibration standards. Based on the gas chromatography data on the conversion of monomers and polymerization degrees of the cleaved side chains determined by the SEC, the ratio of the backbone units with homo- and block copolymer side chains were calculated (Table 1) [44].

The samples of molecular brushes with amphiphilic block copolymer side chains, PI-*g*-(PMAA-*b*-PMMA), hereafter will be labeled as N* and correspond to the numbers of the precursor samples PI-*g*-(PtBMA-*b*-PMMA) in Table 1.

### 2.2. Determination of Molar Mass and Hydrodynamic Characteristics and Investigation of Self-Assembly of Molecular Brushes in Dilute Solutions

As is known, the behavior of amphiphilic polymers in solutions strongly depends on the thermodynamic quality of the solvent with respect to the components. Therefore, the choice of solvents for research is an important task. As can be seen from Table 2, the blocks of the considered brushes (PI, PMAA, PtBMA, PMMA) dissolve in different ways in the solvents we have chosen.

The synthesized molecular brushes (PI-*g*-PtBMA, PI-*g*-(PtBMA-*b*-PMMA) and PI-*g*-(PMAA-*b*-PMMA)) were molecularly dissolved in chloroform, while aggregates were present in dimethylformamide (DMF), tetrahydrofuran (THF) and ethanol. Therefore, their molar mass characteristics were determined in chloroform. The molar masses of the PI macroinitiator were measured in DMF, which is a thermodynamically good solvent for PI.

The solution behavior of copolymers with different compositions was studied by the methods of static (SLS) and dynamic light scattering (DLS) using a Photocor Complex instrument (Photocor Instruments Inc., Russia). The light source was the Photocor-DL diode laser with the wavelength λ = 659.1 nm and controllable power up to 30 mW. The correlation function of the scattered light intensity was obtained using the Photocor-PC2 correlator with 288 channels and processed using the DynalS software. Toluene was used as a calibration liquid, whose absolute scattering intensity *R*_v_ is equal to 1.38 × 10^–5^ cm^–1^. The measurements were conducted at scattering angles θ in the range 45–135°.

In chloroform, the asymmetry of the scattered light was absent for all polymers, even for high-molar mass polymer brushes, therefore, the radii of gyration of scattering objects could not be determined, and their molar masses were obtained by the Debye method. The values of the hydrodynamic radii *R*_h_ were obtained by the DLS method. In the solvents in which the aggregation occurs, we could determine the radii of gyration *R*_g_ and hydrodynamic radius *R*_h_ in those cases when the distribution of the intensity of the scattered light by the particle size was unimodal. Under multimodal distribution, the *R*_h_ value was estimated. Experimental error under determination *R*_h_ is 10%

Prior to the experiments, the scattering cells were rinsed with benzene, evacuated for 15 min and filled with dust-free air. The solutions were prepared at room temperature. All solutions were stored for at least 12 h prior to measurements, in order to ensure a complete equilibration. The investigations were performed in dilute solutions at 21 °C. All solutions were filtered twice into dust-free cells using Chromafil polyamide filters (Macherey-Nagel CmbH&Co.KG, Dueren, Germany) with the pore size of 0.45 µm.

Atomic force microscopy (AFM) (Nanoscope V, Veeco Instruments Inc., Santa Barbara, CA, USA) in tapping mode using commercial tips (NanoSensors, Neuchatel, Switzerland) with a resonance frequency of 300 kHz and a spring constant of ~50 N/m was utilized to characterize the morphology of the molecular brushes. The samples for AFM measurements were prepared by spin casting a chloroform solution of c = 0.1 mg/mL in concentration on a mica surface at 2000 rpm for 1 min. The measurements were carried out in air at room temperature and constant humidity of 55%.

## 3. Results and Discussion

**Figure 2:**
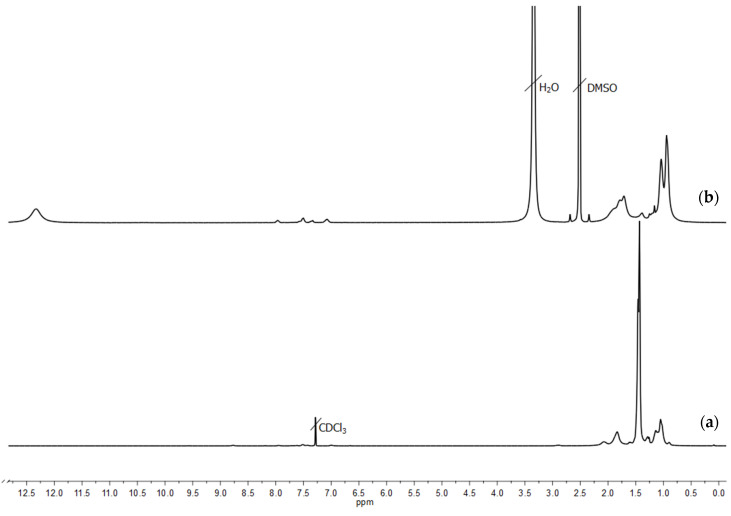
^1^H NMR spectrum of the precursor PI-*g*-PtBMA (**a**) and hydrolyzed PI-*g*-PMAA (**b**) molecular brushes.

**Figure 3:**
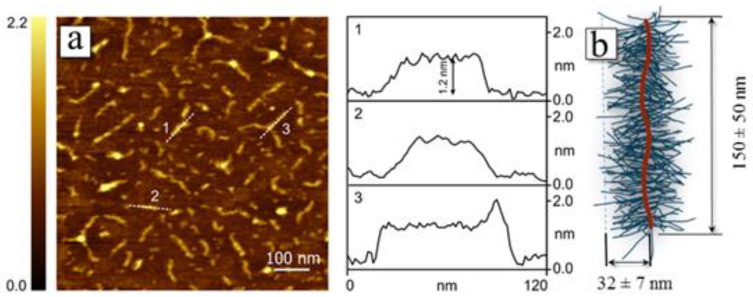
Atomic force microscopy (AFM) micrograph of the PI-*g*-PMAA macromolecules adsorbed on mica substrate (**a**). On the right, the cross-section analysis of the micrograph along the three white dashed lines is shown. The size of the backbone and side chains of PI-*g*-PMAA determined from AFM data (**b**). Degree of polycondensation of the polyimide backbone NPI = 49; degree of polymerization of PtBMA side chains *p* = 130.

The target molecular brushes with amphiphilic block copolymer side chains PMAA-*b*-PMMA were synthesized through formation of an intermediate molecular brush with PtBMA-*b*-PMMA side chains followed by selective hydrolysis of PtBMA chains. This research has focused on three-component molecular brushes with two types of side chains: precursor PtBMA-*b*-PMMA and target PMAA-*b*-PMMA. As described previously in the experimental part, all blocks of the studied molecular brushes (PI, PMAA, PtBMA and PMMA) have different solubility in solvents of different nature. Molecular and conformational characteristics were determined for precursor brushes PI-*g*-(PtBMA-*b*-PMMA) using hydrodynamic methods. The self-assembly behavior in selective solvents was investigated for the target molecular brushes with amphiphilic block copolymer side chains PI-*g*-(PMAA-*b*-PMMA). This approach implies strict consistency between the molar masses of the precursor and amphiphilic side chains. Namely, while preserving the polymerization degree of side chains of molecular brushes, the molar mass of the “inner” block of the side chains of the target brushes should decrease by a factor of ~1.7 compared to the side chains of the precursor brushes due to the hydrolysis of the *tert*-butyl methacrylate units. To prove the validity of this approach, two-component brushes PI-*g*-PtBMA and PI-*g*-PMAA were specially synthesized. The efficiency of the hydrolysis reaction was confirmed by ^1^H NMR spectroscopy. As shown in Figure 2, the characteristic signal of tBMA protons at 1.45 ppm disappears on the spectrum, demonstrating the completeness of hydrolysis. The molar mass and the degree of polymerization of the side chains for PI-*g*-PtBMA precursor brushes were analyzed by SEC. To estimate the chain length of the molecular brushes with poly(methacrylic acid) side chains after selective acidic hydrolysis of precursor molecular brushes, the single macromolecules of PI-*g*-PMAA were visualized using tapping-mode AFM (Figure 3). The chains appear as worm-like structures with the length of side chains of about 32 nm, which corresponds to the degree of polymerization of 128. The obtained results agree well with measurements of precursor side chains’ degree of polymerization obtained by SEC (average polymerization degree of side chains is about 130). This indicates that the polymer-analogous reaction takes place during selective hydrolysis of the precursor brushes with PtBMA-*b*-PMMA side chains.

**Figure 4:**
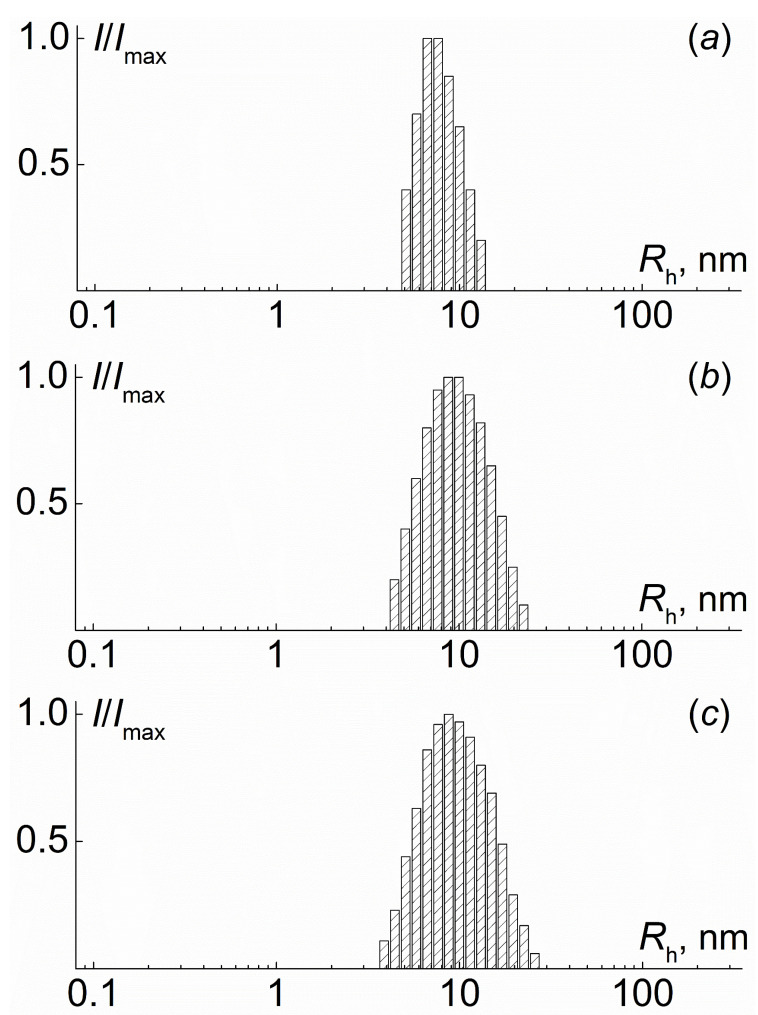
Hydrodynamic radii distribution for chloroform solutions of molecular brushes (Table 1, sample 1) PI-*g*-PtBMA at concentration *c* = 0.054 g·cm^−3^ (**a**), PI-*g*-(PtBMA-*b*-PMMA) at *c* = 0.046 g·cm^−3^ (**b**) and PI-*g*-(PMAA-*b*-PMMA) at *c* = 0.059 g·cm^−3^ (**c**). *I*_max_ is the maximum intensity of scattered light at a given concentration.

**Figure 5:**
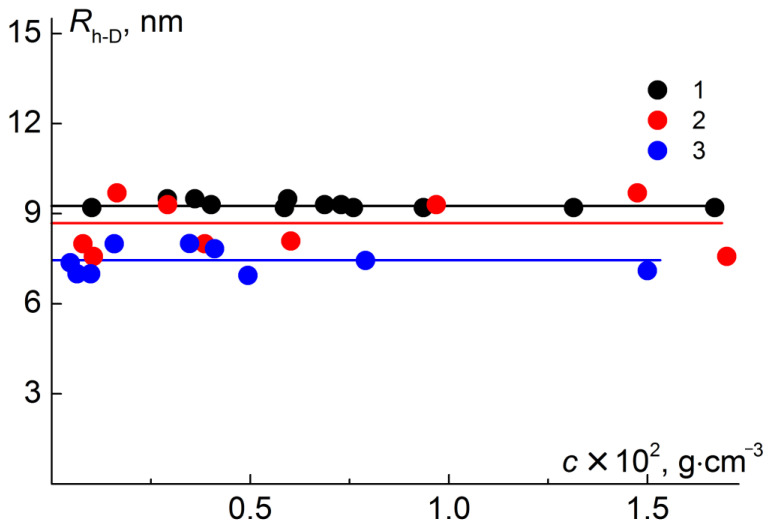
Concentration dependences of hydrodynamic radii for chloroform solution of PI-*g*-(PtBMA-*b*-PMMA) (**1**), PI-*g*-(PMAA-*b*-PMMA) (**2**) and PI-*g*-PtBMA (**3**). Set of copolymers corresponds to sample 1, Table 4.

In chloroform, solutions of the synthesized polymers were molecularly dispersed. As an example, Figure 4 shows the particle size distribution of the intensity of light scattering by solutions of one set of sequentially obtained samples: PI-*g*-PtBMA, PI-*g*-(PtBMA-*b*-PMMA) and PI-*g*-(PMAA-*b*-PMMA) in chloroform. There was no *R*_h_ dependence on concentration for most of the samples (Figure 5), and therefore the concentration-averaged *R*_h_ value was taken as the hydrodynamic radius *R*_h-D_ of the macromolecules. As indicated above, the asymmetry of light scattering for all solutions in chloroform was absent or very small, in order to reliably determine the *R*_g_ values of the macromolecules. The molar masses and hydrodynamic characteristics of the studied samples are presented in Table 3.

It is interesting to compare molar masses for samples in the series of sequentially obtained samples, namely PI, PI-*g*-PtBMA and PI-*g*-(PtBMA-*b*-PMMA), and sample* PI-*g*-(PMAA-*b*-PMMA) (correspond to sample 1 in Table 1) calculated on the basis of SEC data with those obtained by the methods of molecular hydrodynamics and optics. This analysis is given below. The molar mass and hydrodynamic characteristics for polymers of discussed series are presented in Table 3. The PI macroinitiator has a degree of polymerization *N*_PI_ = 37, its number-average molar mass *M*_PI_ = 24,000 g·mol^−1^ and the degree of dispersity *Ð* = 2.8 (Table 1). The molecular brush PI-*g*-PtBMA obtained on this macroinitiator had the molar mass of side chains *M*_s.c._ = 7000 g·mol^−1^ and the grafting density *z* = *x* + *y* = 0.6. Consequently, the number-average molar mass *M*_n_ of the studied copolymer PI-*g*-PtBMA is equal to *M*_n_ = *N*_PI_ × *z* × *M*_s.c._ + *M*_PI_ ≈ 180,000 g·mol^−1^. Considering the dispersity of PI and PtBMA (*Ð* = 1.3) chains, the calculated weight-average molar mass of PI-*g*-PtBMA is from ~500,000 to ~650,000 g·mol^−1^, which agrees well with the value determined by the light scattering method (Table 3). Grafting PMMA chains to PI-*g*-PtBMA, i.e., obtaining the graft copolymer PI-*g*-(PtBMA-*b*-PMMA) with block copolymer side chains, increases the weight-average molar mass of the copolymer by about 380,000 g·mol^−1^. Hence, the calculated molar mass of PI-*g*-(PtBMA-*b*-PMMA) is from 880,000 to 1,030,000 g·mol^−1^, agreeing reasonably with the experimentally determined value for this sample, *M*_w_ = 870,000 g·mol^−1^ (Table 3).

The hydrolysis of PtBMA units is accompanied by a decrease in the *M*_w_ of the copolymer due to the difference in molar masses of the PMAA and PtBMA monomer units. In the case of the series of copolymers under consideration (Table 1, sample 1), the difference between the weight-average molecular masses of the PI-*g*-(PtBMA-*b*-PMMA) and PI-*g*-(PMAA-*b*-PMMA) samples is approximately 190,000 g·mol^−1^, and, accordingly, the calculated molar mass of the target copolymer is from 690,000 to 840,000 g·mol^−1^. The experimental value of molar mass of PI-*g*-(PMAA-*b*-PMMA) is equal to 690,000 g·mol^−1^ (Table 3). Thus, the performed comparison shows good agreement between the molar masses obtained by independent methods.

The conformational analysis for the PI macroinitiator [45] showed that this polymer is a typical flexible-chain polymer with a Kuhn segment length *A* = (1.9–3.0) nm. The obtained value of the hydrodynamic radius *R*_h-D_ for the PI macroinitiator corresponds with good accuracy to a dependence of the type of Mark–Kuhn–Hauwink–Sakurada equation used for the hydrodynamic radius [45].

Unfortunately, due to the small asymmetry of the scattered light, it was impossible to determine the radius of gyration of the studied brushes. Therefore, the conformation of their molecules was estimated by analyzing the values of the hydrodynamic radii of macromolecules. In the transition to graft copolymers, *R*_h-D_ slightly increases (Table 3), being much lower than *R*_h-D_ for PI-*g*-PMMA and PI-*g*-PS [46] in thermodynamically good solvents and close to *R*_h-D_ for PI-*g*-PMMA и PI-*g*-PS in selective solvents (PI-*g*-PMMA and PI-*g*-PS are graft copolymers with a PI backbone and poly(methyl methacrylate) and polystyrene (PS) side chains, respectively) [47]. Therefore, it can be assumed that the conformation of the investigated copolymers in chloroform is close to that for PI-*g*-PMMA and PI-*g*-PS in selective solvents (heptanon-3 and cyclohexane-chloroform mixed solvent in the volume ratio of 99:1), in which a collapsed PI backbone is shielded from the solvent by side chains [47]. Thus, macromolecules of the studied graft copolymers have a core–shell structure with an insoluble solvophobic core surrounded by the solvophilic shell of side chains.

**Figure 6:**
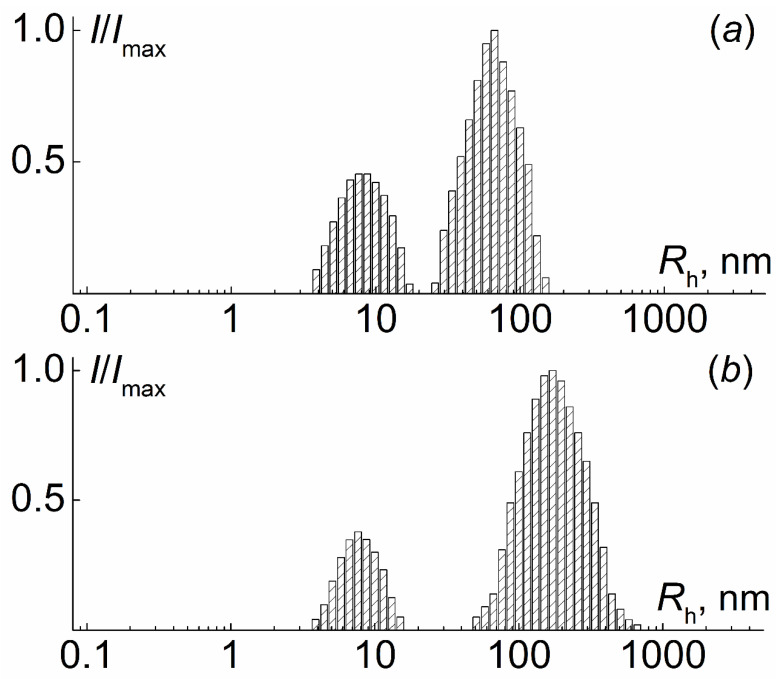
Hydrodynamic radius distribution for PI-*g*-(PMAA-*b*-PMMA) solutions in tetrahydrofuran (THF) at concentration *c* = 0.041 g·cm^−3^ (**a**) and in dimethylformamide (DMF) at *c* = 0.057 g·cm^−3^ (**b**).

**Figure 7:**
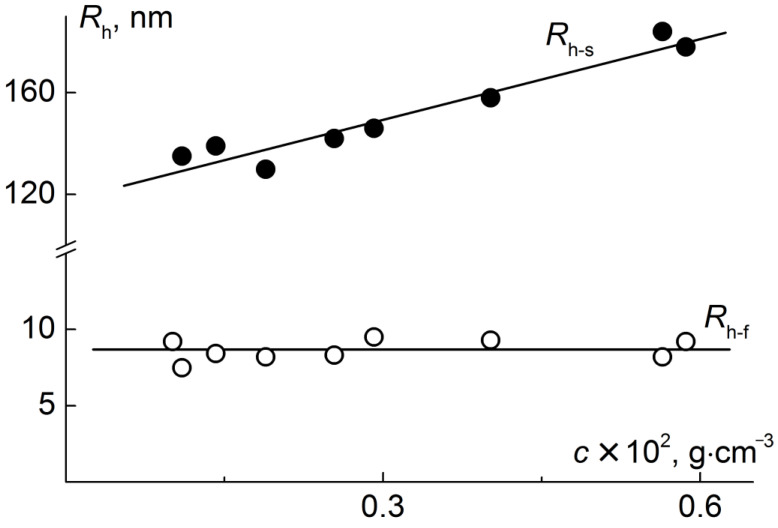
Concentration dependences of hydrodynamic radii for solution of PI-*g*-(PMAA-*b*-PMMA) (Table 4, sample 1*) in DMF.

In other solvents, the target copolymers PI-*g*-(PMAA-*b*-PMMA) were dissolved non-molecularly (Figure 6). In DMF and THF solutions, the hydrodynamic radii distributions were bimodal. Hydrodynamic radii *R*_h-f_, corresponding to the fast mode, did not depend on concentration (Figure 7). Their average value coincided with the radius *R*_h-D_ of the copolymer molecules or was slightly less than *R*_h-D_ (the difference between *R*_h-f_ and *R*_h-D_ did not exceed 20%). This makes it possible to assume that the species responsible for the fast mode are the isolated macromolecules. The observed decrease in molecules size in DMF and THF in comparison with *R*_h-D_ in chloroform can be explained by the worst thermodynamic quality of these solvents and the reduction in macromolecular size caused by compression of the backbone and side chains. The radius of slow-mode particles decreased with dilution, and extrapolation to zero concentration was used to determine the hydrodynamic radii *R*_h-s_ of these species. The particles with radius *R*_h-s_ were aggregates which are formed due to the interactions of the PI backbone and/or PMAA blocks in side chains. Note that the aggregates in DMF are larger than in THF probably due to the worst thermodynamic quality of the latter. The *R*_h-s_ values are on average an order of magnitude higher than the radius *R*_h-D_. Comparing the *R*_h-D_ and *R*_h-s_ values, one can estimate the aggregation degree *z_a_*. Within the framework of the rigid sphere model, *z_a_* = (*R*_h-s_/*R*_h-D_)^3^ (confirmation that the shape of the aggregates is close to spherical is given below). The obtained *z_a_* values lie in the range from 1000 to 3000, while no dependence on the molar mass of the sample and the solvent nature was found.

**Figure 8:**
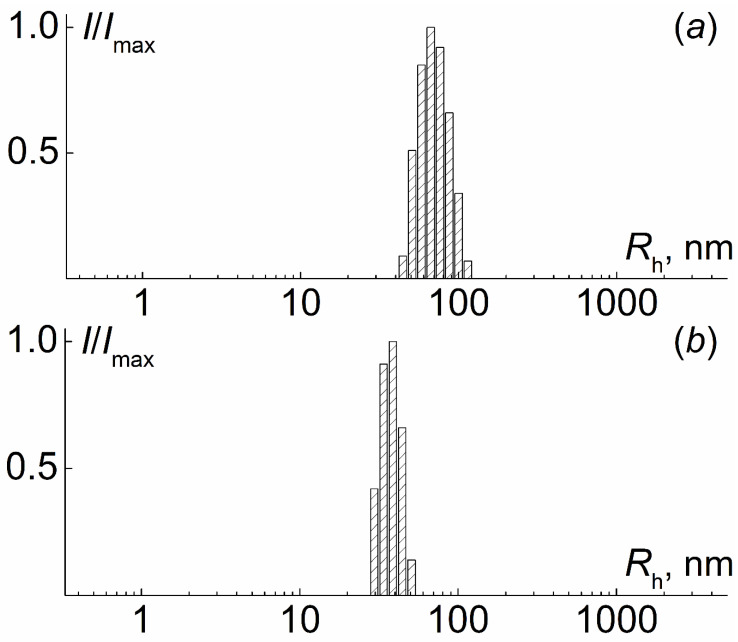
Hydrodynamic radii distribution for samples 1*, Table 1 (**a**) and 3*, Table 1 (**b**) of PI-*g*-(PMAA-*b*-PMMA) in ethanol solution.

**Figure 9:**
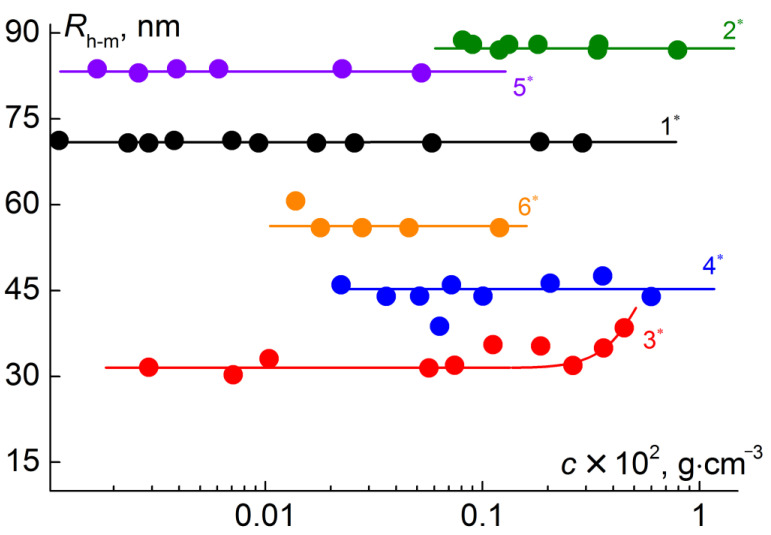
Concentration dependences of hydrodynamic radii *R*_h-m_ for PI-*g*-(PMAA-*b*-PMMA) solution in ethanol. The numbers near the lines correspond to the numbers of the samples* of PI-*g*-(PMAA-*b*-PMMA) in Table 4.

In ethanol solutions of PI-*g*-(PMAA-*b*-PMMA), a unimodal distribution of light scattering intensity on the hydrodynamic radius of scattering particles was observed (Figure 8). As can be seen in Figure 9, the hydrodynamic radii *R*_h-m_ of scattering objects do not depend on the concentration, and their average values, 5 to 10 times exceeding the *R*_h-D_ values, reflect aggregation. An estimate within the framework of a hard sphere model leads to values of the aggregation degree *z_a_* from 150 to 1200. Assumptions about the structure and shape of these aggregates can be made by analyzing the values of the so-called form factor of dissolved particles, namely the ratio *R*_g_*/R*_h-m_ of the radius gyration *R*_g_ and the hydrodynamic radius *R*_h-m_. Note that for the simplest molecular models, an increase in the symmetry of particles leads to a decrease in the form factor. For all investigated solutions of PI-*g*-(PMAA-*b*-PMMA) in ethanol, the ratio *R*_g_*/R*_h-m_ of the gyration radius *R*_g_ to the hydrodynamic radius was lower than 1.1 (Table 4). This makes it possible to suppose that the discussed aggregates had a sufficiently symmetrical shape, and the high intermolecular density is concentrated in the center of the aggregates, i.e., their density decreased with distance from the center to the periphery [48]. Micelle-like structures are formed in ethanol, in which insoluble blocks form their core and PMAA is the shell.

The composition of molecular brushes PI-*g*-(PMAA-*b*-PMMA), in particular the content of PMMA blocks, strongly influenced the aggregate characteristics in ethanol solutions. The decrease in the PMMA fraction in side chains led to a decrease in aggregate size. This fact is illustrated, for example, by a comparison of the *R*_h-m_ and *R*_g_ obtained for samples 3* and 1*, as well as samples 5* and 6* (Table 4). With similar compositions, samples 2* and 6* of PI-*g*-(PMAA-*b*-PMMA) differed in length of the outer PMMA block that leads to a change in the *R*_h-m_ value. In addition, an increase in the *y*/*x* ratio caused a decrease in the *R*_h-m_ radius. Accordingly, aggregates in solutions of sample 3* have the smallest dimensions (Table 4).

## 4. Conclusions

Series of amphiphilic molecular brushes with a polyimide backbone and amphiphilic block copolymer side chains were synthesized by ATRP. Poly(methacrylic acid) was the inner hydrophilic block and poly(methyl methacrylate) was the outer hydrophobic block. The target samples of PI-*g*-(PMAA-*b*-PMMA) differed by structural parameters, namely the grafting density of the side chains, their length and the ratio of hydrophilic and hydrophobic segments in the side chains. The structure and composition of synthesized molecular brushes were confirmed by comparing molar masses of copolymers and their components.

Solution behavior of PI-*g*-(PMAA-*b*-PMMA) samples was determined by their structural parameters and nature of the solvent. In molecularly disperse solutions (in chloroform), the molecules of the synthesized graft copolymers had compact sizes and resembled core–shell structures, the core of which was a collapsed PI backbone and the shell was formed by side chains. Aggregation of macromolecules was observed in most solvents. The aggregate size depended on the thermodynamic quality of the solvent in relation to the copolymer components, as well as on the parameters of the macromolecular architecture. Particularly, in ethanol, an increase in the PMMA content led to a reduction in the aggregate size and an elongation of the outer PMMA block caused a decrease in the hydrodynamic radius. In this solvent, aggregates had a sufficiently symmetrical shape, close to spherical.

## Figures and Tables

**Table 1:** Structural characteristics of molecular brushes with PtBMA-*b*-PMMA side chains.

N	Characteristics of Polyimide Backbone			Characteristics of Side Chains							
	*M_n_* × 10^−3^	*N*_PI_	*Ð*	PtBMA (*x*)		PtBMA-*b*-PMMA (*y*)		Molar Fraction of PI Units with Homo-PtBMA (*x*) and PtBMA-*b*-PMMA (*y*) Side Chains		Average Polymerization Degree of PtBMA (*p*) and PMMA (*q*) Blocks	
				*M_n_* × 10^−3^	*Ð*	*M_n_* × 10^−3^	*Ð*	*x*	*y*	*p*	*q*
1	24	37	2.8	7.0	1.3	37	1.4	0.5	0.1	50	300
2	31	49	2.5	9.8	1.6	11	1.6	0.4	0.6	70	10
3	31	49	2.5	8.6	1.5	25	1.3	0.2	0.8	60	170
4	31	49	2.5	8.6	1.5	10.5	1.5	0.6	0.4	60	20
5	31	49	2.5	8.6	1.5	45	1.3	0.3	0.7	60	360
6	31	49	2.5	8.6	1.5	20	1.7	0.4	0.6	60	110

PI: polymide; PtBMA: poly(*tert*-butyl methacrylate); PMMA: poly(methyl methacrylate); *Ð*: polydispersity index; *M_n_*: number average molar mass; *N*_PI_: degree of polycondensation of the polyimide backbone.

**Table 2:** Solvent characteristics and solubility of the structural elements of molecular brushes.

Solvent	Solvent Characteristics			Solubility of the Structural Elements of Molecular Brushes			
	*ρ*, g × cm^−3^	*η*_0_, cP	*n*_0_	PI	PtBMA	PMAA	PMMA
DMF	0.94	0.80	1.428	+	+	−	+
Chloroform	1.49	0.57	1.446	±	+	−	+
THF	0.89	0.46	1.405	−	+	−	+
Ethanol	0.79	1.08	1.359	−	−	+	−

PMAA: poly(methacrylic acid); DMF: *N*,*N*-dimethylformamide; THF: tetrahydrofuran.

**Table 3:** Molar masses and hydrodynamic characteristics of PI, PI-*g*-PtBMA, PI-*g*-(PtBMA-*b*-PMMA) and PI-*g*-(PMAA-*b*-PMMA) (Table 1, sample 1).

Polymers	Solvents	*M*_w_ × 10^−3^, g·mol^−1^	*R*_h-D_, nm
PI	DMF	39	7.3
PI-*g*-PtBMA	Chloroform	500	7.4
PI-*g*-(PtBMA-*b*-PMMA)	Chloroform	870	9.3
PI-*g*-(PMAA-*b*-PMMA)	Chloroform	690	8.6

**Table 4:** Hydrodynamic characteristics of aggregates in ethanol solutions of polymer brushes PI-*g*-(PMAA-*b*-PMMA).

Samples *	*x*	*y*	*p*	*q*	*R*_h-m_, nm	*R*_g_, nm	*R*_g_/*R*_h-m_
1	0.5	0.1	50	300	70	64	0.9
2	0.4	0.6	70	10	87	81	0.9
3	0.2	0.8	60	170	34	16	0.5
4	0.6	0.4	60	20	45	49	1.1
5	0.3	0.7	60	360	84	56	0.7
6	0.4	0.6	60	110	57	42	0.7

* The numbers of samples in Table 4 correspond to the ones in Table 1.

## Supplementary Materials

The following are available online at https://www.mdpi.com/2073-4360/12/12/2922/s1, Figure S1: Molecular mass distribution for side chains cleaved from molecular brushes with high (a) and low (b) grafting density of the outer PMMA block before and after chain extension.
