# Supplementary material for: Self-Assembly of Molecular Brushes with Polyimide Backbone and Amphiphilic Block Copolymer Side Chains in Selective Solvents
Source: Polymers (Basel). 2020 Dec 5;12(12):2922. doi: 10.3390/polym12122922 (PMC7762168; doi:10.3390/polym12122922)
Supplement: Supplementary file 1 [file polymers-12-02922-s001.pdf]

Supplementary materials

Self-Assembly of Molecular Brushes With Polyimide Backbone And Amphiphilic Block-Copolymer Side Chains In Selective Solvents

Maria Simonova [1,*], Ivan Ivanov [1], Tamara Meleshko [1], Alexey Kopyshev [2], Svetlana Santer [2], Alexander Yakimansky [1] and Alexander Filippov [1]

[1] Institute of Macromolecular Compounds of the Russian Academy of Sciences, Bolshoy pr., 31, Saint Petersburg 199004, Russia; gangspil@gmail.com (I.I.); meleshko@hq.macro.ru (T.M.); kopyshev@uni-potsdam.de (A.K.); santer@uni-potsdam.de (S.S.); yakimansky@yahoo.com (A.Y.); afil@imc.macro.ru (A.F.)

[2] Institute of Physics and Astronomy, University of Potsdam, 14476 Potsdam, Germany; e-mail@e-mail.com

* Correspondence: mariasimonova1983@mail.ru; Tel.: +7-812-328-4102 (M.S.)

Materials:

N-methyl-2-pyrrolidone (98%, Aldrich) and toluene (analytical grade, Vekton, Russia) were dried under heating with anhydrous calcium hydride (99.9%, Aldrich) and distilled in vacuum. Methyl methacrylate (99%, Aldrich) and *tert*-butyl methacrylate (98%, Aldrich) were twice distilled in vacuum prior to use. CuCl (98%, Aldrich) was purified from Cu(II) admixture by washing with glacial acetic acid followed by methanol. Trifluoroacetic acid (99%, Aldrich) was distilled before use. Methylene chloride (reagent grade, Vekton, Russia) and THF (reagent grade, Vekton, Russia) were dried over calcium hydride and distilled. Anisole (99.7%, SigmaAldrich), $FeCl_3 \times 6H_2O$ (97%, Aldrich), 2,2'-bipyridine (bpy) (≥99%, Aldrich), 4,4'-dinonyl-2,2'-bipyridine (dNbpy) (97%, Aldrich), triphenylphosphine (PPh_3) (99%, Aldrich), glucose (≥99.5%, Sigma), Sn(II) 2-ethyl hexanoate ($Sn(EH)_2$) (~95%, Aldrich), *L*-ascorbic acid (≥99.0%, Sigma), and $CuCl_2$ (97%, Aldrich) were used without additional purification. 4,4'-(1,3-Phenylenedioxy) diphthalic anhydride (Ambinter Stock Screening Collections) and 2,4-diaminophenol dihydrochloride (Lancaster, 98%) were dried in vacuum at 140 and 100°C, respectively.

The original PI and the corresponding macroinitiator were synthesized as described in [1]. For the synthesis of grafted copolymers PI-*g*-PtBMA and PI-*g*-(PtBMA-*b*-PMMA) two ATRP procedures (normal ATRP and AGET ATRP) were used [2].

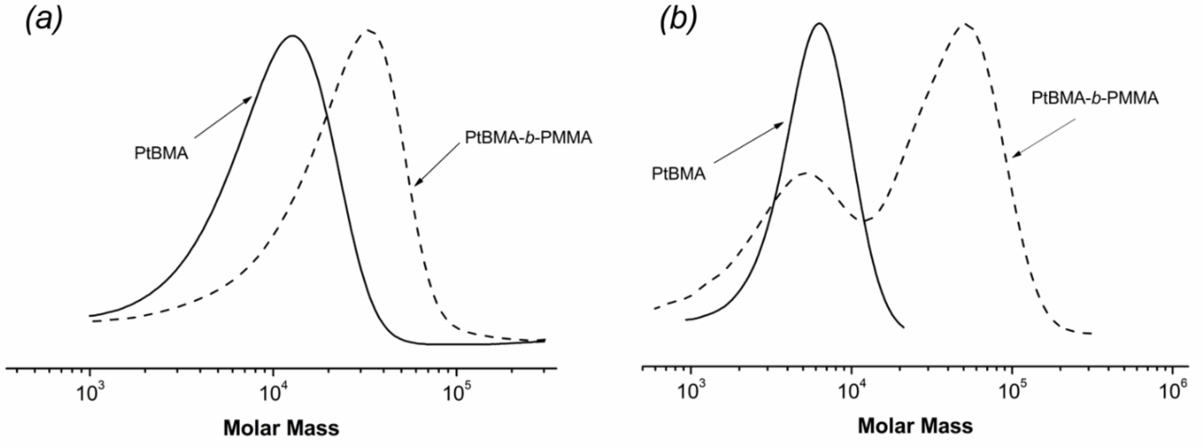

Figure S1. Molecular-mass distribution for side chains cleaved from molecular brushes with high (a) and low (b) grafting density of the outer PMMA block before and after chain extension.
a: PTBMA: M_n = 8600, *Đ* = 1.5; PTBMA–*b*–PMMA: M_n = 24400, *Đ* = 1.3, initiation efficiency = 0.8.
b: PTBMA: M_n = 5500, *Đ* = 1.3; PTBMA–*b*–PMMA: M_n = 37300, *Đ* = 1.3, initiation efficiency = 0.1.

References

1. T. K. Meleshko, D. M. Il'gach, N. N. Bogorad, N. V. Kukarkina, E. N. Vlasova, A. V. Dobrodumov, I. I. Malakhova, N. I. Gorshkov, V. D. Krasikov, and A. V. Yakimanskii, Polym. Sci., Ser. B 52, 589 (2010).
2. Meleshko, T.K.; Ivanov, I.V.; Kashina, A.V.; Bogorad, N.N.; Simonova, M.A.; Zakharova, N.V.; Filippov, A.P.; Yakimansky, A.V. Diphilic macromolecular brushes with a polyimide backbone and poly(methacrylic acid) blocks in side chains. Polym. Sci. Ser. B 2018, 60, 35–50.
